# Exploring patient experiences and acceptability of group vs. individual acupuncture for Cancer-related pain: a qualitative study

**DOI:** 10.1186/s12906-022-03600-6

**Published:** 2022-06-13

**Authors:** Devesh Oberoi, Erica N. Reed, Katherine-Ann Piedalue, Jessa Landmann, Linda E. Carlson

**Affiliations:** 1 Division of Psychosocial Oncology, Department of Oncology, Cumming School of Medicine, University of Calgary, Calgary, AB Canada; 2 Vive Integrative Health Group, 1889 - 45 Street NW, Calgary, AB Canada; 3 Department of Psychosocial Oncology, Cancer Control Alberta, Tom Baker Cancer Centre Holy Cross Site, 2202 2nd St. SW, Calgary, Alberta T2S 3C1 Canada

**Keywords:** Oncology, Acupuncture, Group acupuncture, Individual acupuncture, Qualitative, Patient experience, Social support, Patient privacy

## Abstract

**Background:**

Individual acupuncture (AP) is a safe and effective treatment for cancer-related pain and other symptoms in cancer survivors. However, access to individual AP is limited, and costs can be prohibitive. Group AP could be a more cost-effective alternative as it is less expensive and non-inferior to individual AP for pain relief. Despite growing evidence in favour of group AP, patient acceptability and experience of group AP in cancer patients is relatively unknown. This exploratory study sought to compare patient experiences and acceptability of group versus individual AP in cancer patients.

**Methods:**

Semi-structured, open-ended, in-depth interviews were conducted in a subset of 11 cancer patients enrolled in a randomized non-inferiority trial of group vs. individual AP for cancer pain. Participants for this study were recruited via purposive sampling, aiming for diversity in age, sex, education, employment, cancer types, and treatment arms. Data was analyzed using inductive thematic analysis.

**Results:**

Two major themes were identified: a) overall experience of AP treatment b) value of AP. Participants across both treatment arms acknowledged improvement in pain, quality of sleep, mood and fatigue. Participants in the group AP arm reported a significant increase in perceived social support, while participants in the individual arm valued privacy and one-on-one interaction with the acupuncturist. Although some participants in the group arm had privacy-related concerns before the commencement of the program, these concerns waned after a few AP sessions. Participants across both the treatment arms reported cordial clinician-patient relationship with the acupuncturist. Willingness to pursue AP treatment in the future was comparable across both the treatment arms and was limited by out-of-pocket costs.

**Conclusion:**

Patient acceptability and experience of treatment in group AP was on par with individual AP. Group AP may further augment perceived social support among patients and privacy concerns, if any, subside after a few sessions.

**Trial registration:**

ClinicalTrials.gov (NCT03641222). Registered 10 July 2018 - Retrospectively registered.

**Supplementary Information:**

The online version contains supplementary material available at 10.1186/s12906-022-03600-6.

## Background

Pain is reported by over 50% of cancer patients and survivors, and by two in three palliative cancer patients [1]. Additionally, many cancer patients also experience symptoms of nausea, vomiting, sleep disturbance and fatigue. If untreated, these symptoms can severely impact cancer patients’ quality of life [2, 3]. Conventional cancer treatments do not always reduce the burden of cancer-related symptoms and often have associated side-effects [4]. Currently, the primary treatment for cancer-related pain is opioids, but about 10% of cancer patients who are prescribed opioid medication elect to stop treatments due to adverse effects such as nausea, vomiting and constipation [5]. Moreover, given the current opioid crisis, patients and prescribers are exploring alternatives to avoid long-term use and opioid abuse within cancer care [6].

To help alleviate these negative symptoms and side effects of cancer treatment, patients often seek integrative cancer therapies [7–9]. Acupuncture (AP) is one of the most widely used integrative therapies by cancer patients as an effective method for treating cancer-related symptoms, especially pain management [10–12]. Furthermore, AP has evidence for the management of cancer-related nausea, vomiting, hot flashes, headaches and fatigue [13–15]. Compared to pharmacological treatment, potential side effects of AP are minimal such as minor bruising or bleeding [16], which makes AP a popular treatment choice for cancer patients.

In Canada, AP treatments are primarily delivered on a one-on-one basis. However, access to individual treatment is often limited due to high fees, not usually covered by provincial medical insurance [17]. To increase access to such procedures and to reduce the costs, in the past 10 years, group AP has gained popularity in a range of patient populations across North America [18]. Group AP is a novel method involving up to six patients per room simultaneously receiving treatments, and is a low-cost alternative to individual AP. By decreasing clinic space requirements and increasing the ratio of patients per acupuncturist, the overall patient costs of delivering the treatments are significantly reduced [19–21]. In our previous non-inferiority randomized trial of individual vs. group AP for cancer-related pain [19], group AP was found to be non-inferior to individual AP for alleviating pain as well as for improvement in mood and social support. Moreover, group AP was superior to individual AP for improvement in sleep quality, psychological distress and fatigue [19]. Similar findings have been reported for the efficacy and safety of group AP in other quantitative studies for improvement in cancer-related symptoms such as pain, numbness, digestion and other symptoms [22–24].

Despite growing evidence in favour of group AP in regard to patient acceptability and satisfaction with treatment in various patient populations [20, 21, 25], little is known about its acceptability specifically in cancer populations. A broader understanding is needed to examine the experience and acceptability of group AP compared to individual AP, which goes beyond cost-benefit analyses. The current qualitative study sought to gain insight into patient experiences, perceived effectiveness and acceptability of group vs. individual AP in cancer patients.

## Methods

### Design

This sub-study is a qualitative inquiry using thematic inductive analysis investigating the experiences of a subset of cancer patients enrolled in a randomized non-inferiority trial comparing group vs. individual AP to alleviate cancer pain [19]. The study was approved by the Health Research Ethics Board of Alberta (IRB # HREBA.CC-17-0237) and registered at ClinicalTrials.gov (NCT03641222). The study adherers to the consolidated criteria for reporting qualitative research (COREQ) checklist [26].

### Participants and procedure

Participants in the non-inferiority trial of group vs. individual AP [19] were recruited from the Tom Baker Cancer Centre by referral from the rehabilitation oncology department, or by self-referral between November 2017 and October 2018. Eligible participants for the trial included (1) male or female cancer patients, (2) ≥18 years old, (3) all tumor groups, (4) metastatic or non-metastatic, (5) experiencing pain with a minimum worst pain score (in the previous week) ≥3 on the 10-point Brief Pain Inventory (BPI), (6) willing to be randomized to either condition, and (7) able to attend a minimum of 9 treatment sessions within a 6-week period. Exclusion criteria included the following: (1) AP treatments within the previous 6 months (as it could either underestimate or inflate the effects of the intervention) and (2) a change in cancer treatment that may affect pain control (radiation, systemic therapy, and surgery) within the previous 6 weeks. Participants were randomized to receive either group or individual AP. Participants in both arms (individual and group), had an initial 1-on-1 intake session with the acupuncturist to discuss their health information privately before the sessions started. The treatments were offered twice a week for 6 weeks, for a total of 12 sessions. The sessions for the individual arm lasted approximately 45 minutes. Participants in the group arm were given a 30-minute window to arrive at the centre and were treated on a first-come, first-served basis. Although the group ran for 90 minutes, each participant spent no more than 45 minutes in the group. The treatments were offered during the day and were provided free of cost to the participants. Zero gravity chairs made with woven fabric were used. These are recliner chairs that are specifically designed to suspend the body in a neutral posture where feet are elevated in alignment with the heart. In a reclined position, the person seated on the zero gravity chair experiences a weightless sensation. The chair alleviates pressure points through the spine, back, hips and legs offering a high level of comfort. Traditional Chinese Acupuncture techniques were used wherein every participant had LV3 and LI4 as standard pain points, and then points were chosen on an individualized basis according to location and type of pain. The full study design and the primary results of the trial are published elsewhere [19].

A subset of these trial participants was invited to participate in the current study. In-depth semi-structured open-ended interviews were conducted to gain insight into participants’ experiences of treatment. Participants were selected via purposive sampling to include those from both individual and group AP sessions, both males and females, with varying cancer diagnoses, as well as patients with both good and poor functional status. All participants were contacted by phone.

### Data collection

All interviews were conducted by a female interviewer (ER). At the time of the interviews ER was a honours student who had previous experience conducting interviews and underwent rigorous training. Furthermore, ER had developed a relationship with the participants as she was the study coordinator for the original trial [19]. The interviewer was neither an acupuncturist, a healthcare provider, nor health administrator therefore limiting the risking for any potential biases. The interview guide was developed through an iterative process. An additional file shows the full interview guide and questions. Questions were open-ended and aimed to elicit the experience of AP in the group vs. individual arms, including treatment experiences, social interaction with the acupuncturist and the experience of the physical environment. Follow-up questions were asked to explore changes in pain, mood, and sleep, as well as perceived social support during their time in the study. Further questions explored their initial preference for group or individual AP and their interest in pursuing private AP in the future. Participants who received group AP treatments were asked about their social interactions with other participants in the study and their perceived acceptability of the group. Interviews were conducted one-on-one over the phone or in-person based on participants’ availability and lasted for 20-30 minutes each. At the end of each interview, member validation was done to help improve the accuracy, credibility and validity of the data. The interviewer summarized the information gathered in the interview and restated it to the participants so they could confirm accuracy.

### Data analysis

Interviews were recorded and transcribed verbatim. The data was examined and categorized using inductive thematic analysis [27]. The transcripts were read independently by two researchers to identify patterns in the data. Following familiarisation with the data, inductive coding was used to code the interviews and identify themes. The data was coded in the NVivo 12 software program (QSR International). Labels were generated to summarise each of the transcripts. Splitting (creating more than one separate code from a single initial code), splicing (fusing smaller codes) and linking (clustering smaller codes together around a common theme) were used to develop codes grounded in the data [27]. Codes derived independently were discussed by two researchers (DO, KAP) until consensus was reached. The revised codes were then applied to transcripts. Data collection and preliminary coding ran simultaneously. Data collection ceased when no new themes emerged. After the coding was finished, the researchers looked for patterns in the data using the immersion/crystallization approach [28]. Identification of themes alternated with group reflective analysis until all meaningful themes and patterns in the data were identified and extracted. The findings were consistent with the data and the themes and sub-themes were supported by quotes.

## Results

### Participant characteristics

Six participants from the group arm and five from individual arm were interviewed (*N* = 11). Participants included five males and six females; most participants were either unemployed, retired or were on disability benefits. The majority of participants were married and well-educated. The most common cancer diagnosis was breast cancer, followed by hematological, renal and gynecological. Participants experienced varying intensities and sites of pain at the time of entering the AP treatment. Some of the common pain sites were lower back, neck and knee joint, upper and lower extremities. Participants mentioned the side effects of medication and neuropathy in hands and feet as common causes of pain. The participant characteristics are reported in Table 1.

**Table 1:** Participant characteristics

Participant characteristic	Frequency [N (%)]
Age, mean ± SD 57.1 (±7.92) years	
**Sex**	
Males	5 (45.5)
Females	6 (54.5)
**Employment**	
Employed (Full time/part-time)	4 (36.4)
Retired	1 (9.1)
Unemployed	2 (18.2)
Unable to work due to disability	4 36.4)
**Marital Status**	
Single/divorced	4 (36.4)
Married	7 (63.6)
**Education**	
High school diploma	2 (18.2)
College/Technical school	4 (36.4)
University	5 (45.5)
**Cancer site**	
Breast	5 (45.5)
Appendiceal	1 (9.1)
Uterine	1 (9.1)
Renal	1 (9.1)
Haematological (myeloma, lymphoma, leukemia	3 (27.3)
**Treatment Arm**	
Individual Acupuncture	5 (45.5)
Group Acupuncture	6 (54.5)
**Had prior AP experience**	
Yes	3 (27.3)
No	8 (72.7)

There was no significant difference in the mean number of sessions attended by participants in the individual arm 11.75 (**±**0.5) and the group arm 11.40 (±.89) (*p* = .51). The experiences of AP treatment clustered around two major themes: the general experience of AP treatment, and the value of AP. The major themes were further classified into subthemes.

**Themes**

**Experience of AP treatment**

*Well-organized sessions*

Participants in both AP arms described their experience as “*awesome*,” “*great*,” “*comfortable*,” “*efficient,*” and “*helpful*.” Some of them wished the study could be extended so they could continue to benefit from the AP treatment. Three participants said they would like the study to be more publicized so more patients could learn about the study and benefits of AP in cancer care. Participants acknowledged that the sessions were well-organized. They reported that AP was administered in a calm and soothing environment, with background music and the opportunity to talk with the acupuncturist.

"*Overall, to me, it is quite relaxing and comfortable in these settings. And I got acupuncture previously, years before. I have experienced something like good acupuncture and some bad acupuncture. Somewhere I've had good results, and somewhere I've had bad results, and I found this is the first time I'm doing the AP that is good AP*" (*P130, Group).*

*“I mean, it was well organized; every session was perfectly on time. I'm not used to that kind of you know; usually, I’m sitting in the waiting room just waiting for half a day, right. So, I'm not used to that. So, for me, it was like, wow, everything just happens when it is supposed to happen, this is incredible, right. So, you know I think it was good. I think the facility was good” (P121, Individual).*

One participant expressed dissatisfaction with the timing of the program as it was difficult for her to take time off work twice a week. Some participants suggested that the program could be more efficient by offering free parking, conducting the program at the same site where they received their cancer treatment, and providing more comfortable chairs.

*Rapport with the acupuncturist*

Participants in both group and individual treatment arms stated that they had a great doctor-patient relationship with the acupuncturist. They found the acupuncturist to be “*very kind,” “supportive,” “friendly,” and “sensitive to patients’ concerns*.” Participants added that the acupuncturist’s compassionate and pleasant approach to treatment uplifted their spirits and made a positive impact. They mentioned it was effortless to build a rapport with the acupuncturist as she was trustworthy, and participants felt comfortable disclosing all the information she needed to plan their treatment (Quote 1a, Table 2).

**Table 2:** Themes and supporting quotes

Theme	Subtheme	Quote
**Experience of AP treatment**	Rapport with the acupuncturist	*Quote 1a: “The doctor doing the AP was really good, very friendly, very interested in what my issues were and trying to, I guess, do different things every session to try to see if they could help” P123(Group)*
	Initial preference for the treatment arm	Quote 1b: “*I mean, I will be hooked up to a machine for 6 h, and there will be 20 people around me with the same thing, and it’s interesting to hear their stories, right, you know, because I like to listen to it. But I still fall asleep no matter what. So, to me, I don’t think it would have mattered that way*.” P121 *(Individual)*
	Group vs individual experience	Quote 1c: “*You know you’re already self-conscious about it and then being in a place where they’re going to pull up your shirt a little bit and see it, but that was I think the only negative on a group session*.” P123 *(Group)*
		Quote 1d: “*Well, I haven’t really joined groups to deal with cancer because I feel that if I was around too many people that were really down, you know, really depressed and so on about it that it would make it harder for me to fight it. So, I was -- before I went into it I was hoping for the individual, but once I got into the group nobody was down, nobody was depressed in there, you know*” P122. *(Group)*
**Value of Acupuncture treatment**	Improvement in symptoms	Quote 2a: “*It [the pain] just left, it never went away 100%, but it improved definitely, and it definitely helped me with sleeping, which helps with my overall being in general*.” P130 *(Group)*
	Improvement in pain	*Quote 2b: “I haven’t really noticed any kinds of changes. An average pain level I guess would be about a six”. P116 (Individual)*
	Improvement in mood	Quote 3a: “*Yeah, well, as you can imagine if you’re in that kind of pain all the time you know, it doesn’t matter how upbeat or happy person that you are, pain wears you down, causes your moods to change because it’s just always there, it’s relentless. So I would say I’m definitely more relaxed and easy-going. I’m not so uptight just because I’m not in pain all the time. So definitely, my mood has been elevated*.” P141 *(Group)*
	Improvement in fatigue	Quote 4a: “*Yeah, I still feel that if I did too much before, I would flatline. I felt like I couldn’t even leave the house. Now my fatigue level is to the point where I can get through the day and not feel like I have to crawl up and go to sleep; I can actually stay awake for a whole 18 hours*.” P148 (Group*)*
	Social support	Quote 5a: “*Yeah, I guess so, quite a bit because you know meeting people in the same position that I am and stuff is kind of, yeah, I’m not the only one out there going through this. So, yeah, I’m just a social person, make a difference, yeah.”* P107 *(Group)*
	Appropriate time to receive Acupuncture	Quote 6a: “You’ve *got through the whole process and a very nice -- it’s very nice to have it at the exit when you finish treatment and, it would be nice to have that 6 week period or something, and it’s kind of an end strategy like you’ve done chemo, you’ve done this, you’ve done -- you’re here, your recovery and I don’t know what proportion of people react positively to it but to have that option would be good*.” Participant 126 *(Individual)*
	Intent to pursue private Acupuncture	Quote 7a: “*Yeah, that’s I have no idea what it costs. So, but you know my husband would say go for it, it doesn’t matter what it cost just, go for it because it helps so much. So, yeah*.” P107 *(Group)*
		Quote 7b: “*I think I want to keep doing it on a more continual basis as much as I’d like to. So 35/40 dollars would be more of my budget*.” P130 *(Group)*

*Individual vs. group AP experience
*

- *The initial preference for the treatment arm*

Most participants did not have an initial preference for either group or individual AP at the time of enrolment. Participants were more excited about doing the program and cared less about which treatment arm they would be randomized to. One participant mentioned her preference mainly depended on the timing of the program. Likewise, another participant, who was in the group arm, acknowledged she had no initial preference and was open to doing either group or individual AP in the future. Similar views were echoed by other participants, who did not have an initial choice.

"*I just wanted to be in the study, I really wanted to be -- to be able to participate, so I really didn't have a preference, but I was very happy when I saw the difference, but I'd be happy to go for six people or eight people again in AP*" (P126/*Individual).*

One participant mentioned that preference for individual or group AP depended on individuals’ personality or availability more than anything else. For her, personally, it didn’t make a difference to be in the group arm as she was used to receiving her cancer treatment in the presence of other patients. She added that she enjoyed chatting with people around her and listening to their stories, so the group AP would work best for her (Quote 1b, Table 2).

b. *Group AP* vs *Individual AP experience*

Participants were happy with their experience in the group setting because of the opportunity to socialize with other group members. Some participants randomized to the group arm mentioned they were initially apprehensive about the group setting; however, after finishing the program, they were very satisfied and said, “They would not have it the other way.” These participants reported higher perceived social support compared to those in the individual arm and attributed the change to socializing with other participants.

"*I was actually kind of nervous about going into the group. Just you know I just -- you know I was a little nervous about that, little self-conscious but, no it wasn't like that at all, and I had sort of wanted to be in the individual group but in the end I was glad, I wasn't. And yeah, I guess if I had to do it again, I wouldn't do individual, I'd do groups*." (P141 *Group ).*

One female participant in the group AP said she did not have any initial preference; however, it was comforting that all her group members were females. She added she was not reticent about wearing shorts and T-shirt or about rolling up her pants, which would not be the case if they had men in the same group. A few other participants were not very inclined to join group AP for privacy concerns related to body image. One participant mentioned that she was self-conscious of a scar on her stomach and would be uncomfortable exposing the area in the presence of other patients, therefore she would prefer individual AP (Quote 1c, Table 2).

Some participants were reluctant to join the group AP for fear of being sad and depressed as they anticipated being surrounded by other psychologically distressed cancer patients. However, their perspective changed upon completing group AP. These participants were happier as they had an opportunity to “chit-chat” and socialize with other group members (Quote 1d, Table 2). On the contrary, some participants in the group AP mentioned they were “*not much of a social butterfly*” (P121, Individual) and since they wanted a one-on-one interaction with the acupuncturist, therefore they would prefer individual AP in future. Likewise, one participant said that although she liked talking to people, she was not comfortable discussing her cancer with anybody else, so her preference was strictly individual AP. However, the majority of the participants in the individual sessions reported they were happy with the level of privacy and attention they received from the acupuncturist and would continue to choose individual AP in the future. They were unsure if they would receive equal care in group arm unless they infringed on other patients’ time. One of the participants in the individual AP arm mentioned, she was given the flexibility to reschedule her appointments if she missed those due to work or personal commitments. She added that she received considerable social support from the acupuncturist (Quote 5a, Table 2). Another participant in the individual arm stated that since there were only her and the acupuncturist, it hardly made any difference to her social support.

"*Well, I was in the individual groups, so it was just myself and the doctor*" (*P116, Individual).*

**Value of acupuncture treatment**

Participants in both treatment arms stated that AP offered symptomatic relief for pain as well as other symptoms. One of the participants wanted the (provincial) health department, to integrate AP in standard cancer care as holistic care.

*"I think that moving forward; this will allow for maybe even [Alberta Health Services] to recognize that acupuncture is a large part of the wellness or maintaining wellness or in helping people get well, as you know, as they move forward through the illness. You know these treatments and these drugs are brutal, and if it is [acupuncture treatment] helping to give people a quality of life, then they have to be able to incorporate not just medical care but holistic care as well " (P108, Individual).*

- *Improvement in symptoms*
  i) *Improvement in pain*

The majority of the participants (*n* = 8/11), regardless of the treatment arm, acknowledged moderate to significant improvement in pain following AP treatment in almost all pain sites. Some reported substantial pain relief from a pain score of 10/10 to nearly 0/10 on the pain scale used in the study, while others reported moderate relief, going from a pain score of 6/10 to about 2-3/10. Many participants experienced pain relief lasting for 3-4 weeks to as long as more than 8 weeks post-treatment. A few participants (*n* = 2) stated that their pain relief was short-lived, and pain relapsed either immediately after or within 2 weeks of treatment.

"*And I would say that on the scale of one to ten when I first started going, my pain was probably a seven, seven and a half on an average day. So quite painful, quite debilitating. And I would say that I'm probably down to almost zero on most days. I said to my husband the other day; I can't believe I'm waking up, and I'm pain-free, it's just - it's like a miracle honestly* (P141 G*roup*, Quote 2a Table 2).

Three participants mentioned they did not experience any pain relief following AP treatment, presumably because their pain was longstanding. One participant mentioned she was very optimistic that pain would improve but also considered herself as “*probably one of the worst cases she [acupuncturist] has seen in neuropathy and stuff like that*.” P121. The participant acknowledged some degree of pain relief for a “couple of hours” but admitted that the AP “*unfortunately didn’t help*” (P116, Individual, Quote 2b, Table 2).

ii) *Improvement in mood, sleep, and fatigue*

- *Mood*

Most of the participants (*n* = 9/11) across both treatment arms acknowledged overall improvement in mood. Those who did not experience any changes in mood said they were “*in good spirits going in* “. Others reported changes in mood, ranging from slight to significant improvement. One participant mentioned her anxiety reduced after her third AP session, and she was happier, which was also noticed by her friends and family.

"*Yeah, I kept telling [the acupuncturist], asked her if there was a happiness pin that she could put in. So, for every session, she would put in a couple of happiness pins. I was having difficulty sleeping, and she said I could help you with it*" *(P126, Individual).*

Participants cited several reasons for the improvement in mood, such as relief in pain, an improved outlook, ability to engage in social activities and improved sleep. Some participants mentioned that AP session was an opportunity for them to relax in a “*nice environment, and having a kind and pleasant person to talk to (the acupuncturist) while receiving the treatment was a big factor in uplifting their spirits*.” One participant acknowledged that soon after her AP sessions, she went on anti-depressants and wasn’t sure what the real factor for her improved mood was. But she was grateful that she was experiencing less pain and certainly contributed to her elevated mood level. (Quote 3a, Table 2).

- *Sleep*

Only a few participants reported an improvement in sleep, possibly due to pain relief and lesser fatigue. Most participants (7/11) did not report any improvement or changes in sleep patterns. One participant mentioned she never had any problems related to sleep, so she would not know if the AP has had any effect.

- *Fatigue*

Many participants reported moderate to significant improvement in fatigue and higher energy levels following their AP treatment. Participants said their high energy level lasted longer, and their performance in regard to day to day activities were enhanced. Regardless of the treatment arm, participants attributed their vigour and reduced fatigue to improved sleep quality or pain relief (Quote 4a, Table 2). One participant attributed her improvement in fatigue to energy drinks and her pain medication, which helped her sleep better and added that she wasn’t sure if AP had anything to do with her improvement in fatigue. Two other participants mentioned that they still felt fatigued and had not experienced any significant improvements.

b. *Appropriate time to receive AP*

Most participants across both arms mentioned that they would have preferred to receive AP soon after their cancer treatment. Many of them suggested that AP treatment should be offered immediately after chemotherapy treatments, while others suggested waiting for 4-6 weeks post-chemotherapy. In general, participants anticipated the sooner they could receive AP after their cancer treatment, the more beneficial it would be for them (Quote 6a, Table 2).

Many participants asked for the AP treatment to be offered alongside conventional cancer treatment, such as chemotherapy. One participant mentioned it would be ideal if she could have received AP right after she started experiencing pain, as catching the symptom earlier might have prevented it from worsening.

c. *Intent to pursue private AP in future*

Most participants across both treatment arms said they were interested in continuing their AP treatment after the study completion, as they found AP helpful in reducing their symptom burden. One participant mentioned that she had been “*going to [conventional] treatment after treatment for just the main things*” and would like to go for AP for relief from symptoms occurring as a side effect of cancer treatment. Having already recommended AP treatment to her father, she said she would even recommend it to others.

"*Yes, I look forward -- well when I can afford it, of course. Yes, there's a place -- a traditional Chinese medicine school, students were really great. Fewer instructors, but I've been doing it, and I would like to continue with them*" (P148, Group).

For some participants, the cost of pursuing private AP was a barrier. However, since they found it beneficial, they were willing to pay for the treatment. Participants provided a wide range of out-of-pocket costs they were willing to spend on AP treatment. Some said they would pay up to 20% of the total cost of AP sessions (the other 80% to be covered by private health cover) or a maximum of $35-40 per session. Others said they would pay up to $50 per week and, in some cases, anything ranging between $50-70 sessions. Participants who were willing to pay for AP were optimistic about the benefits of the treatment. One participant said she would be happy to pay up to $75 per session for up to 2 sessions per week. She acknowledged that one-on-one treatment for up to an hour with the acupuncturist, where she would not have to wait at all, would be worth paying that fee. Two participants who were willing to go for private AP said the cost would depend on the cost of other treatments they were undergoing at the time (Quote 7a, and 7b, Table 2).

A few other participants anticipated they would not go for AP treatment on their own because the costs were prohibitive, or they did not find it beneficial. They mentioned that out of pocket costs would be challenging, especially for those who are not working. One participant said that she benefited from AP and would be happy to participate in future studies of AP or in a more extended study where her treatment would cost her nothing. She was, however, averse to going for private AP due to the related costs. Similarly, a few participants who were on disability benefits mentioned they would not be able to afford to pay out of pocket. Participants acknowledged the majority of cancer patients, like themselves, would not have private insurance coverage, and would not be able to afford to pay $80-100 per AP session. One participant mentioned that she would be happy to continue AP treatment if it was part of her standard cancer care plan and was paid for by the health system. Findings suggested that group AP would be a more affordable option for patients willing to pursue private AP in the future.

"*Not everyone plans on spending if you're going twice a month and your cost is 80 dollars or 150, 160 dollars a month, and that's a lot of money to a lot of people, and then you may have to pick time out off your work or have money for transportation or for parking, having a cost too high could really make it impractical for a lot of people especially if they're not working*" *(P130/Group).*

## Discussion

The study explored patient experiences and acceptability of group AP compared to individual AP. Participants across both AP arms valued the treatment experience and were pleased with most of the clinical outcomes that were investigated in this study. Most of the participants, across both treatment arms, were willing to pursue private AP in future, but costs were prohibitive for some participants suggesting that group AP would be a more sustainable option for participants who need to pay for the treatment out of pocket. There were little to no concerns related to privacy or interaction time with the acupuncturist in the group AP arm; instead, most participants described their experience as enjoyable and appreciated the social support they received in the group. A minority of participants felt less comfortable in the group sessions due to self-consciousness or feeling shy or introverted. Participants in the individual arm said they did not have to worry about infringing on other participants’ time with the acupuncturist. Furthermore, they prized the privacy and the flexibility to schedule one-on-one appointments with the acupuncturist. Overall, except for the higher perceived social support in the group AP arm and more privacy in the individual arm, participant experiences and acceptability across the two arms were comparable.

Our findings are consistent with past literature in that the majority of the participants in both group and individual AP valued treatment effects such as pain relief and improvement in mood, sleep, and fatigue [22, 24]. This is consistent with our findings from the original trial and lends further support to the use of group AP, which was found to be non-inferior to individual AP in the treatment of cancer-related pain and cost nearly half of that of individual AP [19].

The high attendance of participants in both group and individual AP was consistent with past studies [29]. Participants in both arms partly attributed their treatment experiences to the well-organized sessions, the flexibility of scheduling appointments (individual AP) as well as to their rapport with the acupuncturist. The research team was concerned that participants in the group AP arm might complain of inadequate attention from the acupuncturist, but the group setting did not hinder or shorten personal interactions between the participants and the acupuncturist. This is in contrast to the findings of a previous study comparing patient experiences, where participants in individual AP sessions described a richer therapeutic relationship with the acupuncturist than those treated in groups [25]. A few group AP participants had minor concerns related to lack of privacy, being overheard, and not receiving enough one on one interaction with the acupuncturist prior to the sessions starting, but for most the worries subsided following the first few sessions. Similar concerns were also noted by Chuang et al., in their qualitative study comparing treatment experiences in the group vs. individual AP [25]. In their study, participants in both treatment arms were concerned about being randomized to the group AP setting; however, in those who were actually assigned to the group AP, such fears were put to rest soon after experiencing the group setting. Like our study, most participants in the group setting were able to achieve pain relief and deep relaxation [25]. Similarly, the benefits of group AP outweighed the concerns for most participants in our study, and some of them acknowledged that they would only go for group AP in the future.

The majority of the participants across both arms experienced moderate to significant pain relief, which also positively impacted their mood, sleep and fatigue. The results corroborate findings from the primary quantitative analyses that found the pain relief was similar across both the treatment arms [19]. The findings are also supported by a previous qualitative study of patients’ experiences of group vs. individual AP, which showed that the experiences related to cancer pain relief were similar across both arms [25]. Participants from both arms advocated for the use of AP sooner rather than later in the course of cancer treatment. Most of them suggested the best time to receive AP would be soon after or a few weeks following completion of chemotherapy. In contrast, some participants suggested AP be administered alongside chemotherapy for early pain relief.

Participants in group AP reported a high level of social support, and their experiences in a group with other cancer patients were engaging and entertaining. In contrast, participants in the individual arm acknowledged no improvement in social support but valued privacy. For the majority of the participants in the group AP sessions, it was comforting to have the opportunity to sit in reclining chairs spaced in a large circle, and to have the choice to either sit quietly or to talk with other patients. Thus, it is important to consider individual patient preferences, such as privacy versus social support, when choosing the best modality to deliver acupuncture.

Most participants, across both arms, expressed interest in pursuing private AP and were willing to spend $35-$75 per session. Although participants valued the benefits of AP, the out of pocket costs were a barrier for some participants, especially those with no income or disability benefits, or with no private health coverage. Participants expressed reservations about paying large amounts (for example, greater than $80 per session) for individual AP sessions, as there would be additional costs for transportation and parking. A minority of the participants stated they would not pursue private AP, as they did not find pain relief or other benefits. This is consistent with past studies [30], which reported that participants did not believe it was worth paying for the private AP if it did not provide any symptomatic relief. While some participants were willing to pay for their private AP treatment, for others, this was not affordable.

The study has several limitations. First, we achieved data saturation with 11 participants in the study, but we did not look for saturation within each treatment arm independently. It is plausible that with a larger sample size within each arm, a few additional differences in the experience of AP might appear. Second, we only sampled participants who completed most of their treatment. The results might have been different if we interviewed those who did not complete a full set of treatments. Third, the study was conducted in an academic tertiary cancer care centre with easy access and a wide array of resources and facilities. It is unknown how well group AP would translate into the resource-limited community settings. Lastly, in our study, both group and individual AP were provided at no cost to participants. It is possible that the requirement to pay for these treatments will affect preference.

## Conclusion

Group AP offers similar treatment experience as individual AP, with the added benefit of increased social support. Group and individual AP were similarly valued for general AP experiences, and also provided similar improvement in symptoms. Individual AP was valued more for privacy, as well as one on one interactions with the acupuncturist and flexibility to schedule appointments, although these were not reported as hindrances by participants in the group arm. Our findings may have relevance for the delivery of AP treatments in resource-limited settings as well as for patients who are limited in their capacity to pay out-of-pocket for AP treatment without compromising the quality of care. Health insurance companies who co-pay for AP treatment would also save money, having to pay lower reimbursements to the patients.

## Supplementary Information

**Additional file 1.** Interview guide

## Data Availability

The datasets used and/or analyzed during the current study are available from the corresponding author on reasonable request.

## Abbreviation

AP: Acupuncture

## References

[1] Warth M, Zöller J, Köhler F (2020). Psychosocial interventions for pain management in advanced cancer patients: a systematic review and meta-analysis. Curr Oncol Rep.

[2] Brandão T, Schulz MS, Matos PM (2017). Psychological adjustment after breast cancer: a systematic review of longitudinal studies: adjustment after breast cancer: a systematic review. Psychooncology.

[3] Wang T, Molassiotis A, Chung BPM (2018). Unmet care needs of advanced cancer patients and their informal caregivers: a systematic review. BMC Palliat Care.

[4] Deng G (2019). Integrative medicine therapies for pain Management in Cancer Patients. Cancer J.

[5] Wiffen PJ, Derry S, Moore RA (2017). Tramadol with or without paracetamol (acetaminophen) for cancer pain. Cochrane Database Syst Rev.

[6] Edlund MJ, Martin BC, Russo JE (2013). The role of opioid prescription in incident opioid abuse and dependence among individuals with chronic non-cancer pain: the role of opioid prescription. Clin J Pain.

[7] Running A, Seright T (2012). Integrative oncology: managing Cancer pain with complementary and alternative therapies. Curr Pain Headache Rep.

[8] Deng G, Cassileth BR (2005). Integrative oncology: complementary therapies for pain, anxiety, and mood disturbance. CA Cancer J Clin.

[9] Qureshi M, Zelinski E, Carlson LE (2018). Cancer and complementary therapies: current trends in survivors’ interest and use. Integr Cancer Ther.

[10] Chiu HY, Hsieh YJ, Tsai PS (2017). Systematic review and meta-analysis of acupuncture to reduce cancer-related pain. Eur J Cancer Care (Engl).

[11] Deng G, Rusch V, Vickers A (2008). Randomized controlled trial of a special acupuncture technique for pain after thoracotomy. J Thorac Cardiovasc Surg.

[12] Lu W, Rosenthal DS (2013). Acupuncture for Cancer pain and related symptoms. Curr Pain Headache Rep.

[13] Pfab F (2006). Acupuncture-point stimulation for chemotherapy-induced nausea and vomiting [Ezzo J, Vickers a et al. J Clin Oncol 2005; 28: 7188–7199]. Dtsch Z Für Akupunkt.

[14] Bao T, Seidman AD, Piulson L (1990). A phase IIA trial of acupuncture to reduce chemotherapy-induced peripheral neuropathy severity during neoadjuvant or adjuvant weekly paclitaxel chemotherapy in breast cancer patients. Eur J Cancer Oxf Engl.

[15] Beer TM, Benavides M, Emmons SL (2010). Acupuncture for hot flashes in patients with prostate Cancer. Urology.

[16] MacPherson H, Thomas K, Walters S (2001). The York acupuncture safety study: prospective survey of 34 000 treatments by traditional acupuncturists. BMJ.

[17] Witt CM, Brinkhaus B, Reinhold T (2006). Efficacy, effectiveness, safety and costs of acupuncture for chronic pain – results of a large research initiative. Acupunct Med.

[18] Chao MT, Tippens KM, Connelly E (2012). Utilization of group-based, community acupuncture clinics: a comparative study with a nationally representative sample of acupuncture users. J Altern Complement Med N Y N.

[19] Reed EN, Landmann J, Oberoi D (2020). Group versus individual acupuncture (AP) for Cancer pain: a randomized noninferiority trial. Evid Based Complement Alternat Med.

[20] Tippens KM, Chao MT, Connelly E (2013). Patient perspectives on care received at community acupuncture clinics: a qualitative thematic analysis. BMC Complement Altern Med.

[21] Asprey A, Paterson C, White A (2012). ‘All in the same boat—: a qualitative study of patients’ attitudes and experiences in group acupuncture clinics. Acupunct Med.

[22] Tofthagen C, Boses S, Healy G (2015). Evaluation of group acupuncture for Cancer-related symptoms: a retrospective analysis. J Palliat Med.

[23] Brick WG, Yaguda SI, Polsky J (2016). Group acupuncture appointments for cancer-related symptoms: a retrospective review of the first year of experience at the Levine Cancer institute. J Clin Oncol.

[24] Kligler B, Nielsen A, Kohrherr C (2018). Acupuncture therapy in a group setting for chronic pain. Pain Med.

[25] Chuang E, Hashai N, Buonora M (2018). “It’s better in a group anyway”: patient experiences of group and individual acupuncture. J Altern Complement Med.

[26] Tong A, Sainsbury P, Craig J (2007). Consolidated criteria for reporting qualitative research (COREQ): a 32-item checklist for interviews and focus groups. Int J Qual Health Care.

[27] Joffe H, Yardley L (2003). Content and thematic analysis.

[28] Crabtree BF, Miller WL (1999). Doing qualitative research.

[29] Simcock R, Fallowfield L, Jenkins V (2009). Group acupuncture to relieve radiation induced Xerostomia: a feasibility study. Acupunct Med.

[30] Bishop FL, Barlow F, Coghlan B (2011). Patients as healthcare consumers in the public and private sectors: a qualitative study of acupuncture in the UK. BMC Health Serv Res.

