# Supplementary material for: Exploring patient experiences and acceptability of group vs. individual acupuncture for Cancer-related pain: a qualitative study
Source: BMC Complement Med Ther. 2022 Jun 13;22:155. doi: 10.1186/s12906-022-03600-6 (PMC9190111; doi:10.1186/s12906-022-03600-6)
Supplement: Supplementary file 1 — Additional file 1. Interview guide [file 12906_2022_3600_MOESM1_ESM.docx]

**Additional file 1: Interview guide**

1. Could you please tell me about your personal experience with the AP sessions?
2. At what point in your cancer journey do you think the treatments would have been the most helpful?
   1. Do you think you received them at the optimal time, or is there a better time?
   2. Do you think it will be better to receive it on treatment, or maybe as soon as treatment ends?
3. Can you tell me about what changes you've noticed in your pain since receiving AP
   1. Can you tell me a little bit more about where your pain originated and about how long were you experiencing pain before you started the sessions
   2. Can you tell me a bit more about what your pain was like before starting the study and then any changes you've noticed since completing the AP?
   3. Have you noticed those changes have kind of lasted, or has your pain gotten better or worse over time?
4. Would you be able to tell me about what changes you've noticed in your mood since receiving AP, if any?
   1. Did those changes start while you're having the AP or kind of after? Somewhere in between?
   2. About how long after did you start noticing those changes?
5. What changes have you noticed in your sleep patterns or fatigue levels since receiving AP? If any?
   1. Did you notice any changes specifically during the sessions like sleep disturbances?
   2. Did you find you're able to sleep longer since then?
6. Did you find any changes during the day in the amount of energy you had or the amount of fatigue you had?
7. Would you be able to comment on the level of social support that you feel you have throughout your time in the AP treatments?
8. Would you be able to comment on the relationship that you have with Jessa [the acupuncturist], with the acupuncturist?
   1. Do you think to have that relationship had an impact on the sessions at all?
9. Before beginning your treatments, did you have a preference for whether you'll receive group or individual AP?
   1. Now, after you've completed your treatments, looking back, how do you feel about being in the other arm (individual sessions versus being in the group sessions or vice versa?
   2. Do you think your experience would have been the same, or would it have been different had you been in the other treatment arm instead?
   3. If you were to enter the study again, what do you think your preference would be? Group or individual?
10. Now that you've done the study, do you think you will continue with AP treatments at all on your own?
    1. If you were to continue with AP treatment, what do you think the maximum you'll be willing to pay for them would be if anything at all?
    2. Do you think that cost is prohibitive to receiving AP?
11. If there's anything else at all you would like to mention about your experience in the study, anything you think would be helpful at all for us to know?
